# α‐Hydroxybutyrate Association With Incident Type 2 Diabetes Over 10 Years Across Different Glycemic States

**DOI:** 10.1111/dom.71155

**Published:** 2026-08-02

**Authors:** Elena Fortin, Anna Norhammar, David Stadler, Peter L. Herzog, Giulia Ferrannini, Ele Ferrannini

**Affiliations:** ^1^ Division of Cardiology, Department of Medicine K2 Karolinska Institutet Stockholm Sweden; ^2^ Center for Diabetes Academic Specialist Center, Region Stockholm Stockholm Sweden; ^3^ Department of Molecular Medicine and Surgery Karolinska Institutet Stockholm Sweden; ^4^ Capio St Görans Hospital Stockholm Sweden; ^5^ DirectSens GmbH Vienna Austria; ^6^ Internal Medicine Unit Södertälje Hospital Södertälje Sweden; ^7^ National Research Council Institute of Clinical Physiology Pisa Italy

**Keywords:** α‐hydroxybutyrate, biomarker, PAROKRANK, Type 2 diabetes

## Abstract

**Aim:**

To investigate the association between baseline plasma α‐hydroxybutyrate (α‐HB), an organic acid derived from aminoacid catabolism and glutathione anabolism, and the 10‐year incidence of Type 2 diabetes (T2DM).

**Materials and Methods:**

This prospective study analysed 1349 participants from the Swedish PAROKRANK study. At baseline, participants without known diabetes underwent a standardised 2‐h oral glucose tolerance test and were categorised into four groups: normoglycemia, impaired fasting glucose, impaired glucose tolerance and T2DM. Incident T2DM was identified over a 9.95‐year median follow‐up via national registries. Plasma α‐HB concentrations were quantified using an enzymatic test kit (XpressGT). Risk of incident T2DM was analysed using uni‐ and multi‐variate Cox regression models. Model performance was further assessed using the category‐less Net Reclassification Improvement (NRI).

**Results:**

α‐HB levels showed a stepwise elevation across the spectrum of dysglycemia at baseline. During follow‐up, 134 (11%) individuals developed T2DM. Each unit increase in log‐transformed α‐HB was associated with a nearly twofold increase in the risk of developing T2DM (HR 1.99; 95% CI 1.32–3.01) in the univariate analysis, which remained significant after adjustment for relevant clinical variables including BMI and fasting Hba1c (HR 1.78; 95% CI 1.19–2.67). The addition of α‐HB to a clinical model yielded a significant NRI improvement (0.23; *p* = 0.01).

**Conclusions:**

Plasma α‐HB is a robust, independent risk marker of incident T2DM over a decade, which identifies long‐term metabolic risk not fully captured by glycaemic status alone. Its reliance on a single fasting measurement may offer a practical tool for refining early risk stratification.

## 1 Introduction

The global prevalence of Type 2 diabetes (T2DM) continues to escalate, imposing a substantial burden on patients and healthcare systems worldwide [1]. Despite advancements in screening, current surveillance strategies—which rely predominantly on glycemic markers, such as HbA_1c_ and fasting glucose—frequently fail to capture the subtle, early transition from normoglycemia to dysglycemia. These markers primarily detect established hyperglycemia, the downstream consequence of disease, rather than the underlying pathophysiology. As a result, many individuals remain undiagnosed until significant insulin resistance has already been present for many years and substantial β‐cell dysfunction has occurred [2, 3]. Consequently, there is an urgent clinical need to identify easily accessible and sensitive circulating biomarkers that reflect the antecedent metabolic perturbations of diabetes, enabling earlier risk stratification and intervention during the prolonged ‘silent’ phase of disease progression.

In the last two decades, metabolomic profiling has identified specific metabolites that precede overt dysglycaemia. Among these, α‐hydroxybutyrate (α‐HB), an organic acid derived from amino acid catabolism (threonine and methionine) and glutathione anabolism also known as 2‐hydroxybutyrate, has emerged as a particularly robust marker of early insulin resistance and dysglycemic states [4, 5]. Mechanistically, α‐HB is generated during increased flux through the hepatic glutathione cycle, in response to rising oxidative stress, elevated fatty acid oxidation and mitochondrial redox imbalance, all conditions characteristic of early insulin resistance [5, 6, 7]. Although the association between α‐HB and prevalent insulin resistance is well documented in cross‐sectional analyses [8, 9], important gaps remain. For instance, long‐term prospective evidence linking α‐HB to incident T2DM is limited and it remains unknown whether α‐HB provides predictive value independent of conventional metabolic risk factors, glycaemic status and measures of insulin resistance.

In this study, we examined the association between baseline plasma α‐HB and 10‐year incidence of T2DM in a well‐characterised cohort stratified by an oral glucose tolerance test (OGTT). We evaluated whether α‐HB predicts T2DM independently of traditional glycaemic markers and insulin resistance as measured by HOMA‐IR, and whether its addition improves risk discrimination beyond established OGTT categories.

## 2 Materials and Methods

### 2.1 Study Design

This is a longitudinal study using the Periodontitis and Its Relationship to Coronary Artery Disease (PAROKRANK) cohort. PAROKRANK was a multi‐center case–control investigation conducted in Sweden to evaluate the association between periodontitis and a first myocardial infarction (MI) [10]. In brief, the original study recruited 805 patients < 75 years of age, hospitalised for a first MI in 17 Swedish hospitals between May 2010 and February 2014, and 805 sex‐ age‐ and area‐matched controls, chosen from the national population registry. Participants with a prior MI, cardiac valve replacement or with any other condition that, in the investigator's opinion, could limit the ability to cope with the protocol were excluded. At study baseline, patients who did not have known diabetes underwent a 2‐h standard OGTT to be categorised as having isolated impaired fasting glucose (IFG), impaired glucose tolerance (IGT) (combined or not with IFG) or newly detected T2DM according to WHO criteria (i.e., fasting glucose ≥ 6.1 mmol/L and 2‐h post‐load glucose (2hPG) ≤ 7.8 mmol/L for isolated IFG, fasting glucose < 7.0 mmol/L and 2hPG 7.8–11.0 mmol/L for IGT and fasting glucose ≥ 7.0 mmol/L and 2hPG ≥ 11.0 mmol/L for T2DM) [11]. Medical information was gathered from hospital records for the patients, whereas it was obtained through standardised interviews for controls. Study visits and laboratory investigations were planned after 6–10 weeks from the hospitalisation for MI patients, and within 10 days thereafter for their matching control.

For the current investigation, a sub‐cohort of the original PAROKRANK without known diabetes (*N* = 146) or missing α‐hydroxybutyrate data (*N* = 70) was investigated. To establish a diabetes‐free baseline cohort for longitudinal analyses, were further excluded those with screen‐detected T2DM at baseline (*n* = 111). Both original cases and controls were followed longitudinally until 31 December 2022, to assess the incidence of T2DM (primary outcome) across different glycemic profiles using International Classification of Diseases (ICD) diagnosis codes and new prescriptions of glucose lowering drugs obtained via linkage with data from the Swedish National Patient Registry, the Cause of Death Registry and the Prescribed Drug Registry, all administered by the Swedish Board of Health and Welfare.

### 2.2 Laboratory Measurements

Venous blood was collected at each hospital during an outpatient visit after overnight fasting and smoking abstinence for at least 12 h and plasma was stored at −70°C in a central biobank at Karolinska Institutet. Biochemical parameters, including complete blood count, lipids (total and high‐density lipoprotein cholesterol and triglycerides), creatinine, fibrinogen, glucose and glycated haemoglobin A_1c_ (HbA_1c_) were analysed locally. Plasma glucose was measured using the validated point‐of‐care system HemoCue Glucose System (HemoCue AB, Ängelholm, Sweden) during a 2‐h OGTT, in the fasting state, and after 30 and 120 min following the ingestion of 75 g of glucose in 200 mL water. High‐sensitivity C‐reactive protein (hs‐CRP) was assayed at a central laboratory (Redhot Diagnostics, Södertälje, Sweden) by means of an ELISA method (MP Biomedicals, New York, USA). Frozen plasma aliquots (6 mL) were sent to Pisa Metabolism laboratory, in Italy, where α‐HB and insulin were assayed. Plasma α‐HB concentrations were measured photometrically with the XpressGT enzymatic test kit in the fasting sample [12]. Insulin concentrations at 0, 30 and 120 min during the OGTT were measured using an electro‐chemiluminescence immune‐assay on a COBAS e411 instrument (Roche, Indianapolis, IN, USA).

### 2.3 Statistical Analysis

Continuous variables are reported as median with interquartile range (IQR) [25th–75th percentile] due to non‐normal distributions; categorical variables are presented as frequency and percentage. Baseline characteristics across tertiles of α‐HB were compared by Kruskal–Wallis tests for independent samples; proportions were compared using a *χ* ^2^ test. Differences in continuous variables across multiple glycemic groups were assessed using the Kruskal–Wallis test. The difference in baseline α‐HB between MI cases and controls was also assessed. Univariate associations between fasting plasma α‐HB and key metabolic parameters (specifically BMI, HbA_1c_, HOMA‐IR and triglycerides) were estimated using Spearman's rank correlation coefficients.

In the longitudinal analysis the primary outcome was incident T2DM, defined as a composite of a new T2DM diagnosis in the outpatient or inpatient specialist register (via ICD codes), or a record in the Medication Registry (ATC codes A10, excluded SGLT2 inhibitors when not combined with another oral glucose lowering medication due to the potential indication for heart failure without diabetes). Participants were censored at the date of diagnosis, death (retrieved via the linkage with the Cause of Death Registry), or end of follow‐up (31 December 2022), whichever occurred first.

Cumulative incidence curves for T2DM across α‐HB tertiles were estimated using the Kaplan–Meier method and compared using the log‐rank test. Cox proportional hazards models were employed to estimate Hazard Ratios (HRs) and 95% Confidence Intervals (CIs) for the association between baseline log transformed α‐HB (per 1‐SD increment and by tertiles) and incident T2DM. Several models were assessed: (1) Model 1: Unadjusted; (2) Model 2: Model 1 further adjusted for age, sex and prior MI; (3) Model 3a: Model 2 further adjusted for BMI and fasting glucose, and Model 3b: Model 2 further adjusted for BMI and HbA_1c_; (4) Model 4: Model 3a further adjusted for 2 h‐PG; (5) Model 5: model 3b further adjusted for HOMA‐IR. Additionally, we formally tested for interaction by including an interaction term between α‐HB and glycemic status as well as prior MI in the final model. As sensitivity analyses, all Cox proportional hazards models were also run in the cases and in the control populations separately. The proportional‐hazards assumption was assessed by Schoenfeld residuals. Statistical significance was considered for a two sided *p* < 0.05.

Incremental model performance was assessed by comparing the base Model 3b (including age, sex, BMI and HbA1c) with an extended model additionally including log‐transformed α‐HB. Predicted absolute risks were estimated at 10 years. Model fit was compared using the likelihood‐ratio test and information criteria, including Akaike's information criterion (AIC) and Bayesian information criterion (BIC). Incremental predictive performance was further assessed using category‐free net reclassification improvement (NRI) and integrated discrimination improvement (IDI). The 95% confidence interval for IDI was estimated using bootstrap resampling with 1000 repetitions. To evaluate clinical usefulness, decision curve analysis (DCA) was performed using 10‐year predicted risks from the base and extended models.

Missingness patterns were explored descriptively. Among variables included in the Cox models, 90% of participants had complete data, with missingness largely restricted to HOMA‐IR (4%) and HbA1c (1%). Logistic regression analyses found no significant associations between missingness and baseline characteristics or incident diabetes, supporting the plausibility of the missing‐at‐random assumption. Given the low proportion of missing data and the absence of clear predictors of missingness, complete‐case analyses were considered appropriate. All analyses were performed using STATA version IC/18.1.

### 2.4 Ethics

The PAROKRANK study was approved by the Regional Ethics Committee at Stockholm and was conducted according to the recommendations outlined in the Declaration of Helsinki. All enrolled participants provided their written informed consent.

## 3 Results

### 3.1 Clinical Characteristics of Study Participants According to Glycaemic Status and α‐HB Tertiles

The final analytical cohort included 1349 individuals (685 controls and 664 cases, Figure S1). Based on the baseline OGTT results, participants were categorised into four glycaemic profiles: normoglycemia (*N* = 899), isolated IFG (*N* = 105), IGT with or without IFG (*N* = 234) and new T2DM (*N* = 111). Baseline characteristics across different glycaemic profiles are summarised in Table 1.

**TABLE 1:** Baseline characteristics of the study population stratified by glycaemic profile.

Variable	Normoglycemia (*N* = 899)	IFG (*N* = 105)	IGT (*N* = 234)	T2DM (*N* = 111)	*p*	Missing
Demographics						
Age, years	63 (56, 67)	64 (59, 68)	65 (60, 69)	65 (60, 68)	< 0.001	
Male sex	730 (81.2%)	91 (86.7%)	186 (79.5%)	92 (82.9%)	0.44	
BMI, kg/m^2^	26.0 (24.0, 28.3)	27.2 (25.0, 29.3)	27.1 (24.5, 29.7)	28.0 (24.9, 31.4)	< 0.001	
Waist circumference, cm	97 (90, 103)	100 (93, 106)	101 (93, 107)	102 (94, 111)	< 0.001	
Marital status					0.77	2
Single	121 (3.5%)	8 (7.6%)	31 (13.2%)	13 (11.7%)		
Married	685 (76.2%)	88 (83.8%)	176 (5.2%)	84 (75.7%)		
Not married	91 (10.1%)	9 (8.6%)	27 (11.5%)	14 (12.6%)		
Education					0.07	5
1–12 years	541 (60.2%)	76 (72.4%)	157 (67.1%)	76 (68.5%)		
> 12 years	353 (39.3%)	29 (27.6%)	75 (32.1%)	35 (31.5%)		
Occupation					0.001	0
Employed	445 (49.5%)	48 (45.7%)	81 (34.6%)	34 (30.6%)		
Unemployed	23 (2.6%)	2 (1.9%)	6 (2.6%)	0 (0.0%)		
Retired	371 (41.3%)	48 (45.7%)	126 (3.8%)	66 (59.5%)		
On sick leave	51 (5.7%)	6 (5.7%)	17 (7.3%)	10 (9.0%)		
Studies/other	3 (0.3%)	0 (0.0%)	3 (1.3%)	0 (0.0%)		
Salary (Swedish kronor/years)					< 0.001	0
180 000–300 000	192 (21.4%)	28 (26.7%)	76 (32.5%)	40 (36.0%)		
< 180 000	93 (10.3%)	14 (13.3%)	28 (12.0%)	19 (17.1%)		
> 300 000	607 (67.5%)	61 (58.1%)	126 (53.8%)	50 (45.0%)		
Clinical history						
Smoking, *n* (%)					0.06	0
Never	383 (42.6%)	40 (38.1%)	77 (32.9%)	41 (36.9%)		
Former (> 1 month)	412 (45.8%)	51 (48.6%)	136 (58.1%)	60 (54.1%)		
Current	97 (10.8%)	12 (11.4%)	21 (9.0%)	9 (8.1%)		
First MI	386 (42.9%)	61 (58.1%)	147 (62.8%)	70 (63.1%)	< 0.001	0
Hypertension, *n* (%)	235 (26.2%)	32 (30.8%)	100 (43.3%)	55 (49.5%)	< 0.001	7
Family history of CVD	281 (36.0%)	31 (36.9%)	67 (32.7%)	32 (32.7%)	0.76	182
Stroke	15 (1.7%)	2 (1.9%)	3 (1.3%)	2 (1.8%)	0.97	5
Peripheral arterial disease	11 (1.2%)	2 (1.9%)	2 (0.9%)	2 (1.8%)	0.82	0
Rheumatic disease	154 (17.4%)	17 (16.7%)	52 (22.3%)	23 (20.7%)	0.31	17
Pulmonary disease	101 (11.4%)	7 (6.9%)	29 (12.5%)	17 (15.3%)	0.27	19
Chronic kidney disease	33 (3.7%)	3 (2.9%)	9 (3.8%)	9 (8.1%)	0.14	0
Depression	81 (9.0%)	9 (8.6%)	16 (6.8%)	10 (9.0%)	0.77	0
Cancer	55 (6.1%)	12 (11.4%)	23 (9.8%)	9 (8.1%)	0.08	0
Biochemical parameters						
Systolic blood pressure, mmHg	130 (120, 140)	130 (120, 140)	135 (120, 149)	138 (120, 150)	0.05	1
Diastolic blood pressure, mmHg	80 (73, 87)	80 (75, 85)	80 (70, 88)	80 (70, 90)	0.94	1
HbA1c, mmol/mol	37 (35, 39)	40 (37, 42)	39 (37, 42)	43 (39, 48)	< 0.001	19
Triglycerides, mmol/L	1.1 (0.8, 1.5)	1.0 (0.8, 1.4)	1.2 (0.9, 1.7)	1.2 (0.9, 1.8)	< 0.001	9
Total cholesterol, mmol/L	4.9 (3.9, 5.8)	4.2 (3.5, 5.1)	4.2 (3.6, 5.4)	4.2 (3.4, 5.2)	< 0.001	7
HDL, mmol/L	1.3 (1.1, 1.6)	1.2 (1.0, 1.5)	1.2 (1.0, 1.5)	1.2 (1.0, 1.6)	< 0.001	8
LDL, mmol/L	2.8 (2.1, 3.7)	2.2 (1.7, 3.2)	2.4 (1.8, 3.2)	2.3 (1.7, 2.9)	< 0.001	23
Hb, g/L	144 (136, 151)	146 (138, 154)	145 (135, 153)	147 (136, 153)	0.31	10
Creatinine, μmol/L	81 (73, 90)	82 (75, 92)	80 (70, 90)	80 (71, 92)	0.51	9
LPK, ×10^9^/L	5.5 (4.7, 6.5)	5.6 (4.6, 6.8)	6.0 (5.1, 7.4)	6.4 (5.3, 7.7)	< 0.001	12
OGTT glucose, mmol/L						
0 min	5.3 (4.9, 5.6)	6.4 (6.3, 6.6)	5.9 (5.5, 6.3)	7.1 (6.4, 7.5)	< 0.001	0
30 min	8.3 (7.4, 9.3)	10 (8.9, 10.9)	9.6 (8.5, 10.8)	11.1 (9.7, 12.5)	< 0.001	5
120 min	5.5 (4.6, 6.4)	6 (5.2, 7.0)	8.8 (8.2, 9.4)			0
hsCRP, mg/L	1.2 (0.6, 2.3)	1.1 (0.5, 2.3)	1.7 (0.8, 3.4)	1.7 (0.8, 3.6)	< 0.001	0
U‐ACR, mg/mmol	1 (0.4, 2.1)	1 (0.5, 2.1)	1 (0.5, 2.6)	1.0 (0.6, 2.7)	< 0.001	501
Thrombocytes, ×10^9^/L	230 (197, 267)	224 (192, 271)	239 (203, 271)	232 (198, 259)	0.10	13
α‐HB, μM/L	37.8 (27.8, 49.5)	43.4 (31.5, 53.2)	43.3 (33.0, 54.3)	49.5 (36.8, 64.0)	< 0.001	0
Medical treatment						
ACE inhibitors	420 (47.0%)	64 (61.5%)	161 (68.8%)	80 (72.1%)	< 0.001	6
ARBs	98 (11.0%)	18 (17.3%)	46 (19.9%)	19 (17.4%)	< 0.001	14
β‐Blockers	391 (43.7%)	59 (56.7%)	156 (66.7%)	68 (61.3%)	< 0.001	5
Diuretics	64 (7.2%)	4 (3.8%)	25 (10.8%)	12 (11.0%)	0.07	10
Calcium channel antagonists	87 (9.7%)	4 (3.8%)	34 (14.7%)	24 (22.0%)	< 0.001	11
Oral anticoagulants	25 (2.8%)	3 (2.9%)	3 (1.3%)	2 (1.8%)	0.58	13
ASA	406 (45.4%)	61 (58.7%)	159 (67.9%)	75 (67.6%)	< 0.001	5
Statins	431 (48.2%)	64 (61.5%)	159 (68.2%)	74 (66.7%)	< 0.001	7
Steroids	29 (3.2%)	1 (1.0%)	13 (5.6%)	5 (4.5%)	0.14	8

*Note:* Data are presented as median (IQR) for continuous measures, and No. (%) for categorical measures.

Abbreviations: α‐HB: α‐hydroxybutyrate; ACE, angiotensin‐converting enzyme; ARB, angiotensin II receptor blocker; ASA, acetylsalicylic acid; BMI, body mass index; CVD, cardiovascular disease; Hb, haemoglobin; HbA1c, glycated haemoglobin; HDL, high‐density lipoprotein cholesterol; hsCRP, high‐sensitivity C‐reactive protein; IFG, impaired fasting glucose; IGT, impaired glucose tolerance; IQR, interquartile range; LDL, low‐density lipoprotein cholesterol; LPK, leukocyte count; OGTT, oral glucose tolerance test; T2DM, Type 2 diabetes mellitus; U‐ACR, Urine Albumin/Creatinine.

**FIGURE 1:**
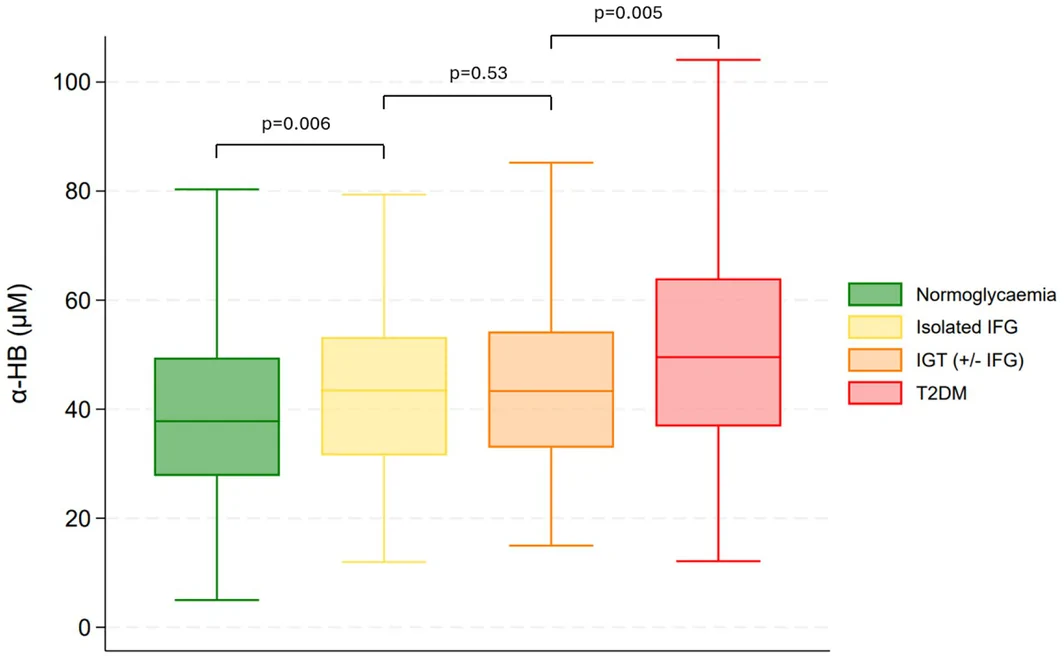
α‐HB levels across the spectrum of dysglycaemia. α‐HB: α‐hydroxybutyrate; IFG: Impaired fasting glucose; IGT: Impaired glucose tolerance; T2DM: Type 2 diabetes mellitus.

α‐HB levels displayed a progressive, stepwise elevation across the spectrum of dysglycemia, as shown in Figure 1. Levels were significantly higher in subjects with IFG compared to normoglycaemia (*p* = 0.006). Levels plateaued between IFG and IGT (*p* = 0.53), before rising significantly again in those with screen‐detected T2DM (*p* = 0.005). α‐HB levels were significantly higher in controls than in MI patients: 40.9 μmol/L (31.1, 52.8) versus 37.3 μmol/L (27.4, 49.4, *p* value < 0.001).

Table 2 reports the baseline characteristics of the study population stratified by α‐HB tertiles, excluding those with newly diagnosed T2DM. Participants in the highest α‐HB tertile (Q3) were significantly more likely to be male (86.9% vs. 75.8%; *p*_overall_ < 0.001) and exhibited a significantly higher BMI (27.1 [24.7, 30.0] vs. 25.6 [23.3, 27.8] kg/m^2^; *p*_overall_ < 0.001) compared to those in the lowest tertile. Metabolic profiling revealed a distinct pattern of dysglycemia associated with elevated α‐HB levels: while baseline HbA_1c_ was similar across tertiles, both fasting and 2hPG rose significantly from the first to the third tertile (both *p*_overall_ < 0.001). Consequently, the prevalence of individuals with IGT rose progressively from 13.1% in Q1 to 23.1% in Q3, whereas the proportion of normoglycemic subjects declined from 80.1% to 65.3%. Regarding lipid metabolism, elevated α‐HB was associated with significantly higher total cholesterol (*p*_overall_ < 0.001) and LDL‐cholesterol (*p*_overall_ < 0.001), while no significant association was observed with HDL‐cholesterol (*p*_overall_ = 0.42). High‐sensitivity C‐reactive protein (hs‐CRP) was also significantly elevated in the upper tertiles (*p*_overall_ < 0.001).

**TABLE 2:** Baseline characteristics across α‐HB tertiles in patients without T2DM.

Variable	α‐HB tertiles			
	Q1 (*N* = 413)	Q2 (*N* = 413)	Q3 (*N* = 412)	*p*
Demographics				
Age, years	63 (56, 68)	64 (58, 67)	63 (57, 67)	0.59
Male sex	313 (75.8%)	336 (81.4%)	358 (86.9%)	< 0.001
BMI, kg/m^2^	25.6 (23.3, 27.8)	26.3 (24.3, 28.7)	27.1 (24.7, 30.0)	< 0.001
Marital status				0.12
Single	62 (15.0%)	47 (11.4%)	51 (12.4%)	
Married	299 (72.4%)	325 (78.7%)	325 (78.9%)	
Not married	51 (12.3%)	41 (9.9%)	35 (8.5%)	
Education				0.07
1–12 years	269 (65.1%)	242 (58.6%)	263 (63.8%)	
> 12 years	144 (34.9%)	166 (40.2%)	147 (35.7%)	
Occupation				0.78
Employed	189 (45.8%)	189 (45.8%)	196 (47.6%)	
Unemployed	10 (2.4%)	10 (2.4%)	11 (2.7%)	
Retired	181 (43.8%)	188 (45.5%)	176 (42.7%)	
On sick leave	28 (6.8%)	21 (5.1%)	25 (6.1%)	
Studies/other	1 (0.2%)	4 (1.0%)	1 (0.2%)	
Salary (Swedish kronor/years)				0.18
180 000–300 000	114 (27.6%)	93 (22.5%)	89 (21.6%)	
< 180 000	52 (12.6%)	38 (9.2%)	45 (10.9%)	
> 300 000	244 (59.1%)	277 (67.1%)	273 (66.3%)	
Clinical history				
Smoking, *n* (%)				0.10
Never	168 (40.7%)	162 (39.2%)	170 (41.3%)	
Former (> 1 month)	188 (45.5%)	202 (48.9%)	209 (50.7%)	
Current	53 (12.8%)	48 (11.6%)	29 (7.0%)	
First MI	231 (55.9%)	187 (45.3%)	176 (42.7%)	
Glycemic group				< 0.001
Normoglycemia	331 (80.1%)	299 (72.4%)	269 (65.3%)	
IFG (isolated)	28 (6.8%)	29 (7.0%)	48 (11.7%)	
IGT (+/− IFG)	54 (13.1%)	85 (20.6%)	95 (23.1%)	
Hypertension, *n* (%)	110 (26.7%)	127 (31.1%)	130 (31.7%)	0.23
Family history of CVD	134 (38.2%)	116 (32.3%)	129 (35.9%)	0.26
Stroke	8 (1.9%)	10 (2.4%)	2 (0.5%)	0.07
Peripheral arterial disease	5 (1.2%)	8 (1.9%)	2 (0.5%)	0.16
Chronic kidney disease	41 (9.9%)	37 (9.0%)	28 (6.8%)	0.26
Cancer	38 (9.2%)	30 (7.3%)	22 (5.3%)	0.10
Biochemical parameters				
HbA_1c_, mmol/mol	38 (35, 40)	38 (35, 40)	38 (35, 40)	0.95
Triglycerides, mmol/L	1.1 (0.8, 1.5)	1.2 (0.9, 1.6)	1.2 (0.9, 1.6)	0.29
Total cholesterol, mmol/L	4.4 (3.7, 5.5)	4.9 (3.8, 5.8)	4.9 (4.0, 5.8)	< 0.001
HDL, mmol/L	1.3 (1.1, 1.6)	1.3 (1.1, 1.6)	1.3 (1.1, 1.6)	0.42
LDL, mmol/L	2.5 (1.9, 3.4)	2.8 (2.0, 3.7)	2.8 (2.1, 3.7)	< 0.001
Creatinine, μmol/L	81 (72, 91)	82 (72, 92)	80 (73, 88)	0.31
OGTT glucose, mmol/L				
0 min	5.4 (5.0, 5.8)	5.5 (5.1, 5.9)	5.5 (5.1, 6.0)	< 0.001
120 min	5.6 (4.6, 6.8)	6.0 (4.9, 7.3)	6.6 (5.3, 7.7)	< 0.001
hsCRP, mg/L	1.2 (0.6, 2.1)	1.2 (0.6, 2.4)	1.5 (0.8, 2.8)	< 0.001
α‐HB μmol/L	25.6 (21.0, 29.2)	38.9 (35.7, 42.6)	56.8 (51.2, 67.2)	< 0.001
Medical treatment				
ACE inhibitors	231 (56.1%)	209 (50.9%)	205 (50.1%)	0.18
ARBs	48 (11.7%)	59 (14.4%)	55 (13.5%)	0.52
β‐Blockers	228 (55.3%)	194 (47.1%)	184 (45.0%)	0.01
Diuretics	31 (7.5%)	28 (6.8%)	34 (8.3%)	0.72
Calcium channel antagonists	37 (9.0%)	49 (12.0%)	39 (9.5%)	0.33
Oral anticoagulants	12 (2.9%)	12 (2.9%)	7 (1.7%)	0.44
ASA	236 (57.3%)	198 (48.2%)	192 (46.8%)	< 0.001
Statins	244 (59.5%)	211 (51.3%)	199 (48.5%)	0.01
Steroids	13 (3.1%)	16 (3.9%)	14 (3.4%)	0.93

*Note:* Data are presented as *n* (%) or median (IQR), as appropriate. If not otherwise stated, cases data were retrieved ‘at follow‐up’, 6–10 weeks after their myocardial infarction.

Abbreviations: α‐HB: α‐hydroxybutyrate; ACE, angiotensin‐converting enzyme; ARB, angiotensin II receptor blocker; ASA, acetylsalicylic acid; BMI, body mass index; CVD, cardiovascular disease; Hb, haemoglobin; HbA1c, glycated haemoglobin; HDL, high‐density lipoprotein cholesterol; hsCRP, high‐sensitivity C‐reactive protein; IFG, impaired fasting glucose; IGT, impaired glucose tolerance; IQR, interquartile range; LDL, low‐density lipoprotein cholesterol; LPK, leukocyte count; OGTT, oral glucose tolerance test; T2DM, Type 2 diabetes mellitus; U‐ACR, Urine Albumin/Creatinine.

Notably, a history of prior MI was most frequent in the lowest α‐HB tertile (55.9% in Q1 vs. 42.7% in Q3; *p*_overall_ < 0.001). In line with this finding, the use of cardioprotective medications, specifically β‐Blockers, ASA and statins, followed an inverse distribution, being most prevalent among subjects in the lowest α‐HB tertile, likely reflecting the study design and the more intensive pharmacological management required for subjects with established cardiovascular disease at baseline.

### 3.2 Correlations Between α‐HB Levels and Metabolic Variables

In correlation analyses (Table 3), plasma α‐HB levels showed significant positive correlations with key metabolic markers. Specifically, α‐HB was positively correlated with 2‐h post‐load glucose (*r* = 0.22, *p* < 0.001), HOMA‐IR (*r* = 0.16, *p* < 0.001) and BMI (*r* = 0.19, *p* < 0.001). No significant correlation was observed between α‐HB and HbA_1c_ (*r* = −0.01, *p* = 0.63).

**TABLE 3:** Spearmans correlations between α‐HB and metabolic factors.

	rho
HbA_1c_	−0.1
Fasting glucose	0.07
2 h Post‐load glucose	0.22^a^
HOMA‐IR	0.16^a^
BMI	0.19^a^
Triglycerides	0.05
HDL colesterol	0.01
Total colesterol	0.12^a^

Abbreviations: α‐HB: α‐hydroxybutyrate; BMI: body mass index; HbA1c: glycated haemoglobin A1c; HDL: high‐density lipoprotein; HOMA‐IR: homeostatic model assessment of insulin resistance.

^a^ *p* value < 0.01.

### 3.3 Associations Between α‐HB and Incident T2DM During Follow‐Up

Follow‐up data were available for 1224 included study participants. Among these, 134 patients developed T2DM during a median follow‐up time of 9.95 years (IQR: 9.06–10.87 years), corresponding to an event rate of 11.60 (95% CI 9.86–13.68) per 1000 patient‐years.

**FIGURE 2:**
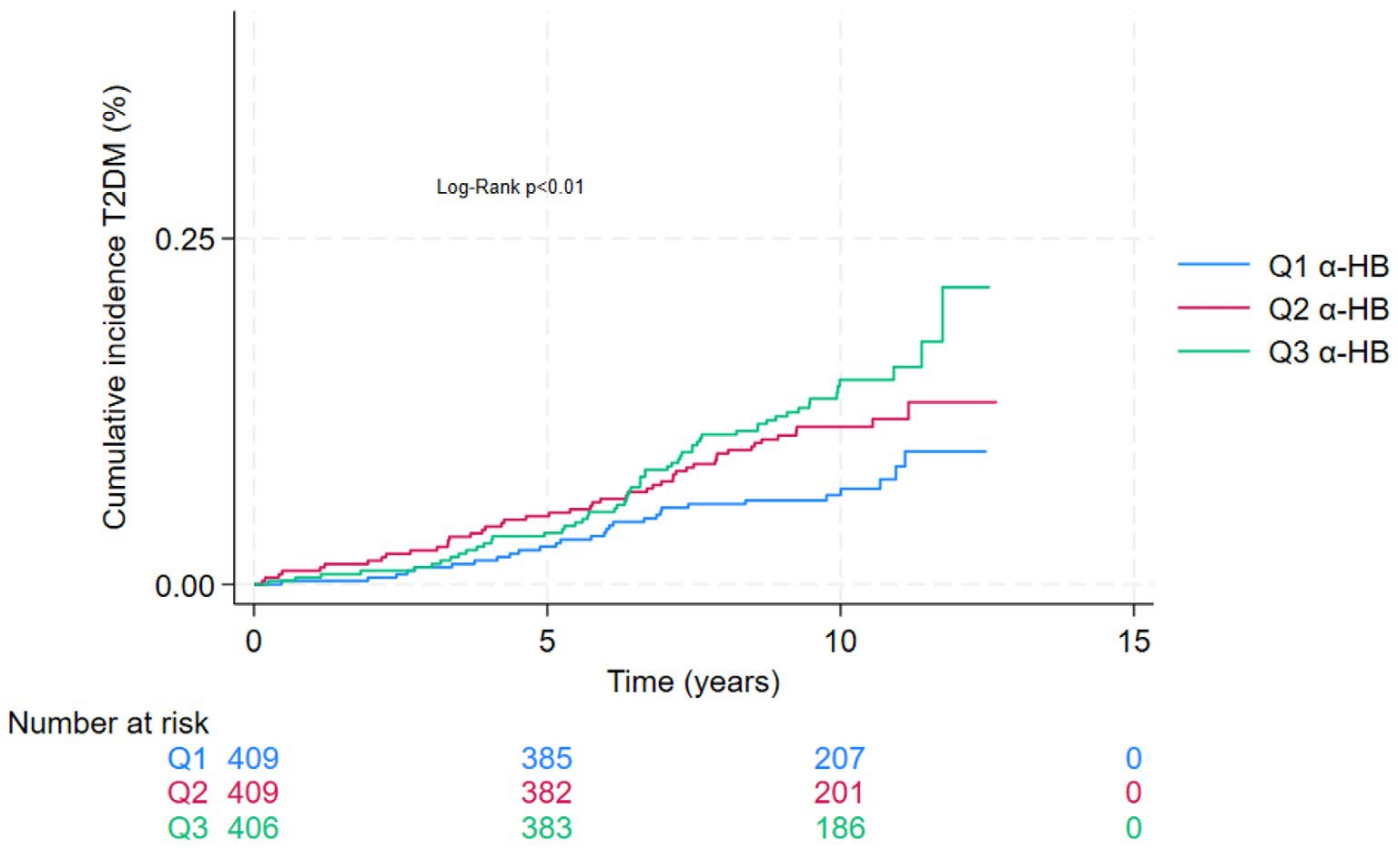
Incidence of Type 2 diabetes by α‐hydroxybutyrate levels. Kaplan–Meier estimates of diabetes incidence over 10 years. Log‐rank *p* < 0.001. α‐HB: α‐hydroxybutyrate.

Kaplan–Meier survival analysis revealed a graded association between baseline α‐HB levels and the incidence of T2DM (Figure 2). The cumulative incidence of T2DM significantly differed across tertiles (Log‐rank *p* < 0.01), with the highest incidence observed in the top tertile and the lowest in the bottom quartile. In Cox regression analysis, individuals in the highest tertile had a more than two‐fold increased risk of developing T2DM (HR 2.06; 95% CI 1.31–3.21; *p* = 0.002). A significant elevation in risk was already apparent in the second tertile (HR 1.63; 95% CI 1.03–2.60; *p* = 0.04).

**FIGURE 3:**
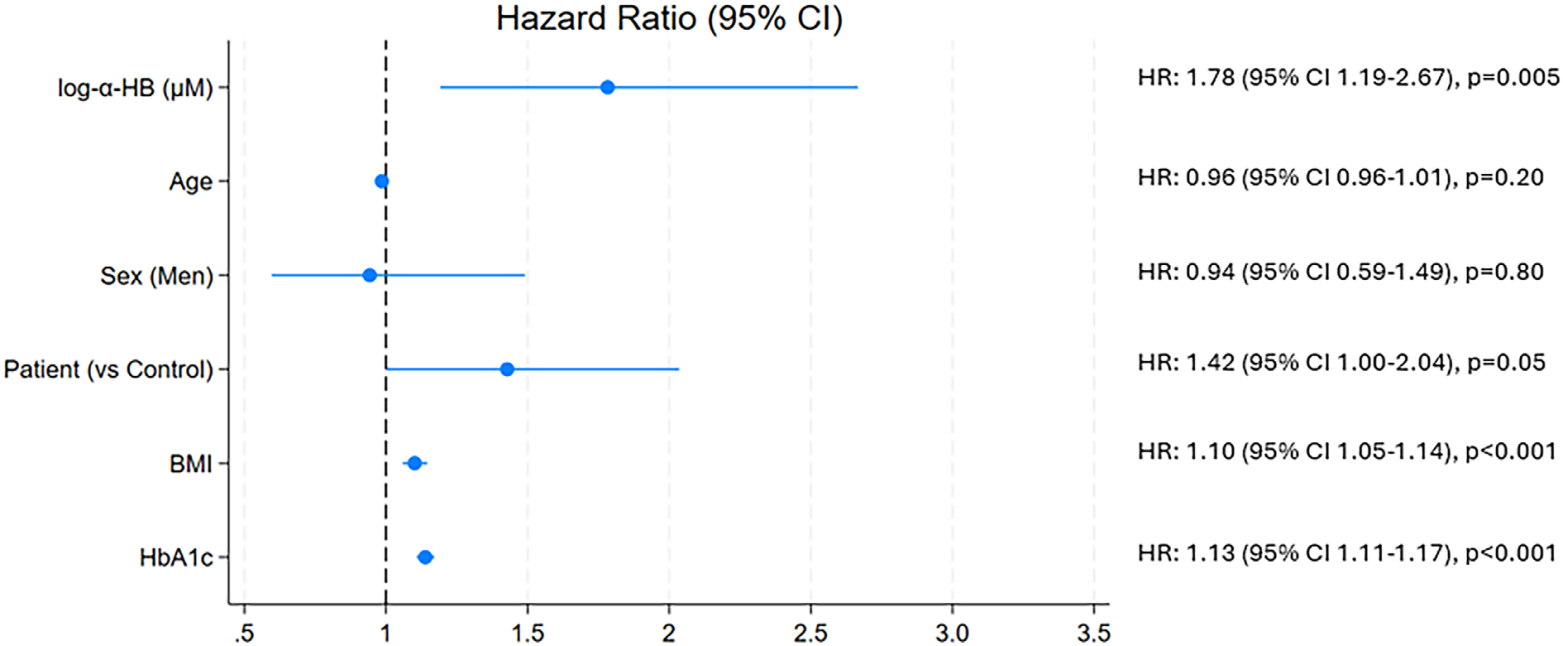
Forest plot of adjusted Hazard Ratios for incident T2DM (Model 3b). Forest plot displaying Hazard Ratios (HR) and 95% Confidence Intervals (CI) derived from a multi‐variable Cox proportional hazards regression model (Model 3b). Blue markers indicate the point estimate for the HR, and horizontal error bars represent the 95% CI. α‐HB: α‐hydroxybutyrate; BMI: Body mass index; HbA1c: Glycated haemoglobin A1c.

When analysed as a continuous variable, log‐transformed α‐HB levels remained significantly associated with incident T2DM. Each unit increase in log‐transformed α‐HB was associated with a nearly twofold increase in the risk of developing T2DM (HR 1.99; 95% CI 1.32–3.01; *p* = 0.001). In the multi‐variate Cox regression model, after adjusting for age, sex and prior MI (Model 2) α‐HB remained significantly associated with an increased risk of incident T2DM (HR 2.11 per unit increase in log α‐HB; 95% CI 1.40–3.17). This association remained statistically significant even after additional adjustment for baseline BMI and fasting glucose (Model 3a; HR 1.72; 95% CI 1.11–2.66) or baseline BMI and HbA_1c_ (Model 3b; HR 1.78; 95% CI 1.19–2.67; Figure 3). In the latter model, each 1‐unit increase in BMI was associated with a 10% increase in the hazard of incident T2DM (HR 1.10, 95% CI 1.05–1.14), and each 1‐unit increase in HbA_1c_ was associated with a 13% increased hazard (HR 1.14, 95% CI 1.11–1.17). Finally, the association between α‐HB and incident T2DM persisted even after adjusting for HOMA‐IR (Model 5; HR 1.74; 95% CI 1.14–2.67), although it was attenuated and lost statistical significance when 2‐h post‐load glucose was added (Model 4; HR 1.33; 95% CI 0.85–2.07). Estimates of different models are summarised in Table S1.

There was no significant interaction between α‐HB and baseline glycemic status, or prior MI status, regarding T2DM risk (*p*_interaction_ > 0.05).

In the Cox models restricted to controls, point estimates were of the same magnitude, although Model 3a, Model 4 and Model 5 fell short of statistical significance, as summarised in Table S2. Similarly, in cases, in Model 4 the association with incident T2DM was not statistically significant, whereas it was in the other models, with similar HRs (Table S3).

The addition of log‐transformed α‐HB improved model fit compared with the base clinical model. The likelihood‐ratio test supported improved fit of the extended model (*χ* ^2^ = 7.46, *p* = 0.0063). The extended model also had a lower AIC than the base model (1737.9 vs. 1743.4), while BIC was slightly lower (1763.4 vs. 1763.8). Incremental predictive performance was supported by a category‐free NRI of 0.23 and an IDI of 0.0113 (bootstrap 95% CI 0.0002–0.0371), indicating a modest improvement in discrimination. In DCA, both models provided positive net benefit across the evaluated range of 10‐year risk thresholds (Figure S2).

To assess clinical utility, we evaluated model discrimination. The base model including age, sex, BMI and HbA_1c_ yielded a C‐statistic of 0.76. The addition of α‐HB to this model did not result in a significant improvement in the C‐statistic (ΔAUC = 0.007; *p* = 0.31); however, it did yield a significant category‐less NRI of 0.23 (95% CI 0.05–0.41; *p* = 0.01). Among those who developed diabetes, 57.5% had their predicted risk correctly increased by the addition of α‐HB, whereas among those who remained healthy, 52.8% had their predicted risk correctly decreased.

## 4 Conclusions

The present prospective analysis of a well‐characterised cohort with baseline OGTT phenotyping and 10 years of follow‐up provides evidence that plasma α‐HB is an independent, long‐term marker of risk for incident T2DM. The association found remained robust after adjustment for age, sex, BMI, fasting glucose, HbA_1c_ and HOMA‐IR, and was only attenuated after inclusion of 2‐h post‐load glucose. Importantly, α‐HB improved risk reclassification despite minimal change in global discrimination metrics. Collectively, these findings indicate that α‐HB captures an early metabolic signal that precedes overt diabetes by nearly a decade and was associated with modest improvement in risk reclassification beyond conventional glycemic markers.

Our results extend prior cross‐sectional observations identifying α‐HB as a marker of insulin resistance and IGT by providing long‐term prospective validation. In the RISC cohort, α‐HB correlated with insulin sensitivity measured by the hyperinsulinemic–euglycemic clamp [13], supporting its role as an early marker of impaired insulin action. Other earlier metabolomic studies identified increasing α‐HB levels as one of the earliest detectable metabolic shifts in the transition to glucose intolerance [4, 7, 8, 12]. However, longitudinal data is limited. Our 10‐year prospective data confirm that α‐HB's value is not merely a reflection of current glycemia but captures an underlying metabolic perturbation that precedes clinically manifest T2DM by a decade. The observed graded increase in α‐HB concentrations across glycemic categories supports the concept that α‐HB reflects progressive metabolic deterioration. Notably, the absence of a significant rise between IFG and IGT suggests that α‐HB may preferentially track early hepatic metabolic stress rather than peripheral glucose disposal defects [14, 15]. This differs from previous observations by Cobb et al. [4], who reported elevated α‐HB specifically in IGT, but such discrepancies may derive from differences in baseline metabolic health of the participants, the degree of underlying peripheral versus hepatic insulin resistance, as well as the phenotypic overlap between IFG and IGT. The persistence of the association after adjustment for HOMA‐IR further indicates that α‐HB captures aspects of metabolic dysfunction not fully reflected by static measures of insulin and glucose. HOMA‐IR primarily reflects hepatic and fasting insulin sensitivity rather than peripheral insulin sensitivity, consistent with the observed attenuation of the association specifically by 2hPG, which more closely reflects peripheral glucose disposal. In this context, α‐HB may represent a biochemical marker of impaired metabolic flexibility, an early defect in substrate handling that precedes frank hyperglycaemia.

The biological plausibility of α‐HB as a T2DM risk marker is grounded in its role in redox metabolism. As proposed by Gall et al., while α‐HB is a recognised marker of increased glutathione stress [16], its elevation also reflects a shift in hepatic redox state and mitochondrial substrate handling. In early insulin resistance, hepatic fatty acid oversupply increases β‐oxidation, raising the mitochondrial NADH/NAD^+^ ratio. This redox shift alters the activity of various dehydrogenases within the tricarboxylic acid cycle and, via the action of lactate dehydrogenase (LDH) isoforms, favours the conversion of α‐ketobutyrate to α‐HB [17]. Thus, elevated α‐HB likely reflects integrated disturbances in oxidative stress, redox balance and mitochondrial substrate handling.

A key finding of the present study is that α‐HB remained independently associated with incident T2DM after adjustments for HbA_1c_ and fasting glucose, whereas further adjustment for 2 h‐PG attenuated this association and statistical significance was no longer achieved. Analyses conducted in cases and controls separately confirmed this finding. Notably, 2hPG has consistently demonstrated stronger prognostic performance for incident T2DM than fasting glucose or HbA_1c_ alone [18, 19], reflecting its ability to detect early impairments in postprandial glucose disposal and peripheral insulin resistance. The loss of significance after 2hPG adjustment indicates that α‐HB and 2hPG may capture a shared component of early post‐load metabolic inefficiency instead of being independent. Unlike the OGTT, however, which requires a multi‐hour protocol and serial sampling, α‐HB offers the distinct advantage of requiring only a single measurement. This potential clinical utility is supported by recent evidence showing that fasting α‐HB can identify individuals with postprandial dysglycemia despite normal fasting glucose and HbA1c, supporting its role as a pragmatic screening biomarker beyond conventional fasting measures [20]. Moreover, evidence suggests that α‐HB levels remain stable over the time course of an OGTT, indicating its potential utility in non‐fasting settings [21]. This stability significantly enhances feasibility for practicing physicians, and the implementation of measuring α‐HB in clinical routine may reduce discomfort for the patients while also facilitating the selection of individuals where the more time‐ and resource consuming confirmatory post‐load testing is warranted.

Interestingly, α‐HB concentrations at baseline were generally higher in men than in women; however, the longitudinal association between rising α‐HB and metabolic deterioration was consistent after sex adjustment.

Having established the biological plausibility of α‐HB, we also extensively assessed whether this translated into incremental predictive value beyond conventional risk factors. There was a modest but consistent incremental predictive value beyond established clinical risk factors, suggesting that α‐HB provides metabolic information that HbA_1c_ (as a measure of average glycemia) might not capture at the sub‐clinical level. From a precision prevention standpoint, such refinement could facilitate earlier implementation of lifestyle or pharmacological interventions.

### 4.1 Strengths and Limitations

This study has several important strengths. First, the comprehensive metabolic characterisation at baseline, including standardised OGTT phenotyping, minimised misclassification bias and strengthened internal validity. Second, the nearly 10‐year follow‐up enabled assessment of long‐term T2DM risk rather than short‐term metabolic fluctuation. Third, the availability of detailed clinical and metabolic covariates allowed extensive multi‐variable adjustment, including hepatic insulin resistance as measured by HOMA‐IR and post‐load glycemia, permitting a stringent evaluation of the independent contribution of α‐HB. Participants in the lowest α‐HB tertile were more likely to have had a prior MI and to receive intensive cardioprotective therapy, likely reflecting selection dynamics within PAROKRANK's original case–control design. However, no interaction was observed between prior myocardial infarction and the association between α‐HB and incident T2DM, suggesting that the metabolic risk signal conveyed by α‐HB operates independently of overt cardiovascular disease. Fourth, the present findings gain additional clinical relevance in light of recent evidence demonstrating that enzymatically measured α‐HB can identify postprandial dysglycemia in individuals with normal fasting glucose and HbA1c [20], supporting the utility of α‐HB across different stages of dysglycemia, from early detection of metabolic abnormalities to long‐term prediction of incident T2DM.

Several limitations should be acknowledged. The Swedish cohort and the original case–control design may limit generalizability to other populations. The marked male predominance of the cohort (> 80%) limited statistical power for sex‐stratified analyses. All models were adjusted for sex and the association was consistent after adjustment, but sex‐specific predictive performance could not be reliably established, and our findings may be less generalizable to women. As this was a post hoc analysis, the results should be interpreted as exploratory. Additionally, incident T2DM was identified through linkage with national health registries and while registry‐based ascertainment ensures near‐complete follow‐up, it may underestimate true incidence due to undiagnosed or untreated cases. To mitigate this limitation, we cross‐linked multiple registries, including the national prescribed drug register, and excluded SGLT2 inhibitors prescribed for non‐diabetic indications. Another concern is that α‐HB was measured only at a single baseline time point, precluding assessment of intra‐individual variability or longitudinal changes. Future studies incorporating serial metabolomic profiling may clarify whether dynamic changes in α‐HB further refine risk prediction. Finally, as with all observational studies, causality cannot be inferred. Although extensive multi‐variable adjustment was performed, residual confounding by unmeasured metabolic or lifestyle factors cannot be entirely excluded.

In conclusion, plasma α‐HB is a robust, independent risk marker of incident T2DM over 10 years. By reflecting early hepatic redox imbalance and oxidative stress, α‐HB captures a long‐term metabolic risk signal not fully represented by conventional glycaemic measures. Although external validation is required before clinical implementation, and while it does not outperform the diagnostic accuracy of the OGTT, its reliance on a single fasting measurement may represent a promising candidate for early risk stratification in routine clinical settings.

## Author Contributions

**Elena Fortin:** data curation, formal analysis, visualisation, writing – original draft. **Anna Norhammar:** investigation, supervision, project administration, writing – review and editing. **David Stadler:** resources, investigation, writing – review and editing. **Peter L. Herzog:** resources, investigation, writing – review and editing. **Giulia Ferrannini:** supervision, formal analysis, writing – review and editing. **Ele Ferrannini:** conceptualization, formal analysis, supervision, writing – review and editing.

## Funding

This work was supported in an in‐aid grant by DirectSens GmbH, Vienna, Austria, to prof. Ele Ferrannini's institution. The PAROKRANK study was academically driven and was supported by grants from AFA Insurance Foundation, the Swedish Heart‐Lung Foundation, the Swedish Research Council, the Swedish Society of Medicine and the Stockholm County Council.

## Conflicts of Interest

D.S. and P.L.H. are employees of DirectSens GmbH, Vienna, Austria. E. Ferrannini has received an in‐aid grant for the present work. The other authors declare no conflicts of interest.

## Supporting information


**Table S1:** Estimates of the correlation between α‐HB and incident T2DM in different models in the whole population.
**Table S2:** Estimates of the correlation between α‐HB and incident T2DM in different models in controls.
**Table S3:** Estimates of the correlation between α‐HB and incident T2DM in different models in cases.

**Figure S1:** Flowchart of study population selection and glycaemic classification. IFG: impaired fasting glucose; IGT: impaired glucose tolerance; MI: myocardial infarction; OGTT: oral glucose tolerance test; T2DM: Type 2 diabetes mellitus.

**Figure S2:** Decision curve analysis.

## Data Availability

Data are available from the corresponding author upon reasonable request and under the Karolinska Institutet data sharing agreement.
