# Supplementary material for: α‐Hydroxybutyrate Association With Incident Type 2 Diabetes Over 10 Years Across Different Glycemic States
Source: Diabetes Obes Metab. 2026 Aug 2;28(10):9493–504. doi: 10.1111/dom.71155 (PMC13538760; doi:10.1111/dom.71155)
Supplement: Supplementary file 1 [file DOM-28-9493-s003.docx]

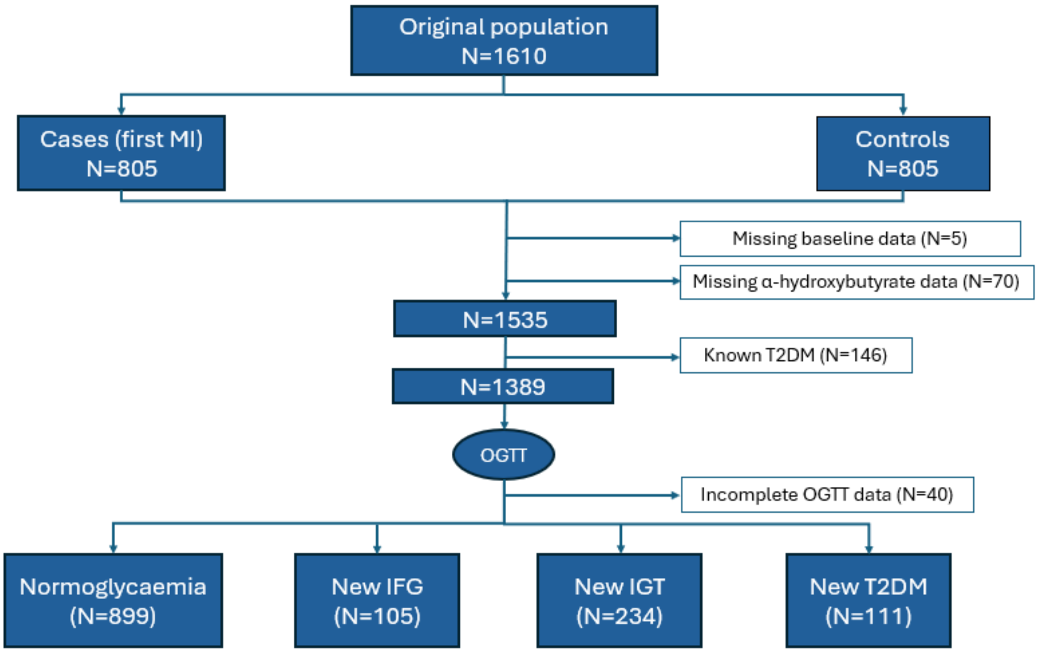
Original population
N=1610
Cases (first MI)
N=805
Controls
N=805
Missing baseline data (N=5)
Missing α-hydroxybutyrate data (N=70)
N=1535
Known T2DM (N=146)
N=1389
OGTT
Incomplete OGTT data (N=40)
Normoglycaemia
(N=899)
New IFG
(N=105)
New IGT
(N=234)
New T2DM
(N=111)
