# Supplementary material for: α‐Hydroxybutyrate Association With Incident Type 2 Diabetes Over 10 Years Across Different Glycemic States
Source: Diabetes Obes Metab. 2026 Aug 2;28(10):9493–504. doi: 10.1111/dom.71155 (PMC13538760; doi:10.1111/dom.71155)
Supplement: Supplementary file 1 [file DOM-28-9493-s001.docx]

**Supplemental Table 1**. Estimates of the correlation between α-HB and incident T2DM in different models in the whole population.

| **Model** | **HR, 95% CI** | **P value** | **Model details** |
| --- | --- | --- | --- |
| Model 1 | 1.99 (1.32–3.01) | 0.01 | Unadjusted |
| Model 2 | 2.11 (1.40–3.17) | <0.0001 | Model 1 further adjusted for age, sex and prior MI |
| Model 3a | 1.72 (1.11–2.66) | 0.014 | Model 2 further adjusted for BMI and fasting glucose |
| Model 3b | 1.78 (1.19–2.67) | 0.005 | Model 2 further adjusted for BMI and HbA_1c_ |
| Model 4 | 1.33 (0.85–2.07) | 0.21 | Model 3a further adjusted for 2h-PG |
| Model 5 | 1.74 (1.14–2.67) | 0.010 | Model 3b further adjusted for HOMA-IR |

Abbreviations: 2h-PG: 2-hour postload glucose; BMI: body mass index; CI: confidence interval; HbA1c; glycated haemoglobin A1c; HOMA-IR: homeostasis model assessment for insulin resistance; HR: hazard ratio.

**Supplemental Table 2**. Estimates of the correlation between α-HB and incident T2DM in different models in controls.

| **Model** | **HR, 95% CI** | **P value** | **Model details** |
| --- | --- | --- | --- |
| Model 1 | 2.55 (1.26–5.14) | 0.009 | Unadjusted |
| Model 2 | 2.46 (1.21–4.99) | 0.013 | Model 1 further adjusted for age, sex and prior MI |
| Model 3a | 1.53 (0.73–3.23) | 0.261 | Model 2 further adjusted for BMI and fasting glucose |
| Model 3b | 2.19 (1.05–4.56) | 0.036 | Model 2 further adjusted for BMI and HbA_1c_ |
| Model 4 | 1.17 (0.54–2.53) | 0.689 | Model 3a further adjusted for 2h-PG |
| Model 5 | 1.33 (0.62–2.87) | 0.461 | Model 3b further adjusted for HOMA-IR |

Abbreviations: 2h-PG: 2-hour postload glucose; BMI: body mass index; CI: confidence interval; HbA1c; glycated haemoglobin A1c; HOMA-IR: homeostasis model assessment for insulin resistance; HR: hazard ratio.

**Supplemental Table 3**. Estimates of the correlation between α-HB and incident T2DM in different models in cases.

| **Model** | **HR, 95% CI** | **P value** | **Model details** |
| --- | --- | --- | --- |
| Model 1 | 1.89 (1.14–3.11) | 0.013 | Unadjusted |
| Model 2 | 1.94 (1.18–3.20) | 0.009 | Model 1 further adjusted for age, sex and prior MI |
| Model 3a | 1.71 (0.99–2.93) | 0.053 | Model 2 further adjusted for BMI and fasting glucose |
| Model 3b | 1.67 (1.01–2.76) | 0.046 | Model 2 further adjusted for BMI and HbA_1c_ |
| Model 4 | 1.35 (0.77–2.35) | 0.291 | Model 3a further adjusted for 2h-PG |
| Model 5 | 1.80 (1.06–3.04) | 0.029 | Model 3b further adjusted for HOMA-IR |

Abbreviations: 2h-PG: 2-hour postload glucose; BMI: body mass index; CI: confidence interval; HbA1c; glycated haemoglobin A1c; HOMA-IR: homeostasis model assessment for insulin resistance; HR: hazard ratio.
